# Crystal structure of 3,14-diethyl-2,13-di­aza-6,17-diazo­niatri­cyclo­[16.4.0.0^7,12^]docosane dinitrate dihydrate from synchrotron X-ray data

**DOI:** 10.1107/S2056989019007655

**Published:** 2019-05-31

**Authors:** Dohyun Moon, Sunghwan Jeon, Keon Sang Ryoo, Jong-Ha Choi

**Affiliations:** a Beamline Department, Pohang Accelerator Laboratory, Pohang 37673, Republic of Korea; b Department of Chemistry, Andong National University, Andong 36729, Republic of Korea

**Keywords:** crystal structure, protonated macrocycle, nitrate, hydrate, hydrogen bonds, synchrotron radiation

## Abstract

The crystal structure of title salt, C_22_H_46_N_4_^2+^·2NO_3_^−^·2H_2_O, has been determined using synchrotron radiation at 220 K. The structure determination reveals that protonation has occurred at diagonally opposite amine N atoms. The asymmetric unit contains half a centrosymmetric dication, one nitrate anion and one water mol­ecule. The mol­ecular dication, C_22_H_46_N_4_^2+^, together with the nitrate anion and hydrate water mol­ecule are involved in an extensive range of hydrogen bonds. The mol­ecule is stabilized, as is the conformation of the dication, by forming inter­molecular N—H⋯O, O—H⋯O, together with intra­molecular N—H⋯N hydrogen bonds.

## Abstract 2

In the title salt, C_22_H_46_N_4_^2+^·2NO_3_^−^·2H_2_O, the dication lies about an inversion center. In the crystal, N—H⋯O, O—H⋯O and N—H⋯N hydrogen bonds connect the anions, cations and water mol­ecules, forming a three-dimensional network.

## Chemical context   

The 3,14-diethyl-2,6,13,17-tetra­aza­tri­cyclo­(16.4.0.0^7,12^)doco­sane macrocycle (C_22_H_44_N_4_, *L*) contains a cyclam backbone with two cyclo­hexane subunits. Ethyl groups are also attached to the 3 and 14 carbon atoms of the propyl chains that bridge opposite pairs of N atoms in the structure. The macrocyclic ligand *L* is a strongly basic amine capable of forming the dication, [C_22_H_46_N_4_]^2+^ or the tetra­cation [C_22_H_48_N_4_]^4+^ in which all of the N—H bonds are generally available for hydrogen-bond formation. These di- or tetra-ammonium cations may be suitable for the removal of toxic heavy metal ions from water. The crystal structures of [Cu(*L*)](ClO_4_)_2_ (Lim *et al.*, 2006 ▸), [Cu(*L*)](NO_3_)_2_, [Cu(*L*)(H_2_O)_2_](SCN)_2_ (Choi *et al.*, 2012 ▸), [Ni(*L*)(NO_3_)_2_] (Subhan & Choi, 2014 ▸), [Ni(*L*)(N_3_)_2_] (Lim *et al.*, 2015 ▸) and [Ni(*L*)(NCS)_2_] (Lim & Choi, 2017 ▸) have been reported. In these complexes, Cu^II^ or Ni^II^ cations have tetra­gonally distorted octa­hedral environments with the four N atoms of the macrocyclic ligand in equatorial positions and the O/N atoms of anions or water mol­ecules in the axial positions, while [Ni(*L*)](ClO_4_)_2_·2H_2_O (Subhan & Choi, 2014 ▸) has a square-planar geometry around the Ni^II^ atom that binds to the four nitro­gen atoms of the macrocyclic ligand. The macrocyclic ligands in the Cu^II^ and Ni^II^ complexes adopt the most stable *trans*-III conformation. Recently, we also reported the crystal structures of [C_22_H_46_N_4_](ClO_4_)_2_ (Aree *et al.*, 2018 ▸), [C_22_H_46_N_4_]Cl_2_·4H_2_O (Moon *et al.*, 2013 ▸) and (C_22_H_44_N_4_)_2_·2NaClO_4_ (Aree *et al.*, 2018 ▸). To further investigate the hydrogen-bonding behavior, we report here on the synthesis of a new hydrated nitrate salt, [C_22_H_46_N_4_](NO_3_)_2_·2H_2_O, (I), and its structural characterization by synchrotron single-crystal X-ray diffraction.
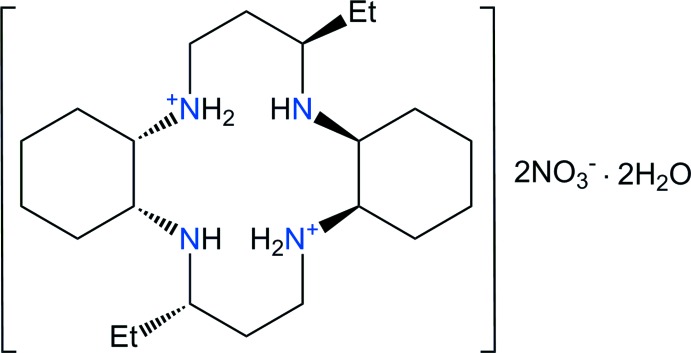


## Structural commentary   

**Figure 1:**
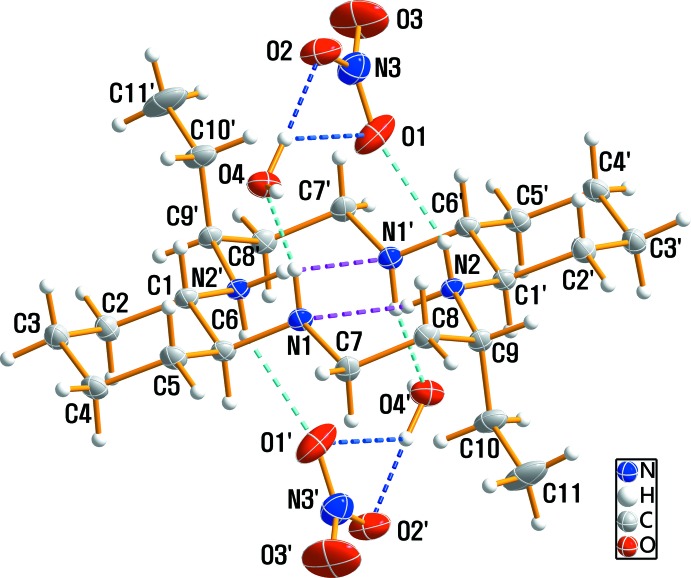
The mol­ecular structure of (I), drawn with displacement ellipsoids at the 30% probability level. Primed atoms are related by the symmetry code (1 − *x*, 1 − *y*, 1 − *z*). Dashed lines represent N—H⋯O (cyan), N—H⋯N (pink) and O—H⋯O (blue) hydrogen-bonding inter­actions, respectively.

The title compound has a positively charged macrocyclic dication, two nitrate anions and two solvent water mol­ecules and was prepared during a study of the macrocyclic ligand and its silver(II) complex. An ellipsoid plot of the mol­ecular components in compound (I) is shown in Fig. 1 ▸ along with the atom-numbering scheme. The asymmetric unit consists of one half of the macrocycle, which lies about a center of inversion, one nitrate anion and one solvent water mol­ecule. The four N atoms are coplanar, and the two ethyl substituents are *anti* with respect to the macrocyclic plane as a result of the mol­ecular inversion symmetry. The six-membered cyclo­hexane ring is in a stable chair conformation. Within the centrosymmetric diprotonated amine unit [C_22_H_46_N_4_]^2+^, the C—C and N—C bond lengths vary from 1.517 (2) to 1.533 (2) Å and from 1.485 (2) to 1.501 (2) Å, respectively. The macrocycle is protonated at the N2 atom, which is similar to the situation found for [C_22_H_46_N_4_](ClO_4_)_2_ (Aree *et al.*, 2018 ▸), but differs from the protonation of the N1 atom in [C_22_H_46_N_4_]Cl_2_·4H_2_O (Moon *et al.*, 2013 ▸). The protonation on the N atom might depend on the location of the acceptor atoms of the counter-anion involved in hydrogen bonding. The ranges of N—C—C and C—N—C angles are 108.07 (11) to 111.14 (12)° and 115.09 (11) to 115.19 (10)°, respectively. The bond lengths and angles within the [C_22_H_46_N_4_]^2+^ dication are comparable to those found in [C_22_H_46_N_4_](ClO_4_)_2_ (Aree *et al.*, 2018 ▸) and [C_22_H_46_N_4_]Cl_2_·4H_2_O (Moon *et al.*, 2013 ▸). The nitrate counter-anion has a distorted trigonal-planar geometry as a result of the influence of hydrogen bonding on the N—O bond lengths and the O—N—O angles. The N—O bond distances range from 1.204 (3) to 1.214 (2) Å and the O—N—O angles from 117.4 (2) to 123.1 (3)°.

## Supra­molecular features   

**Figure 2:**
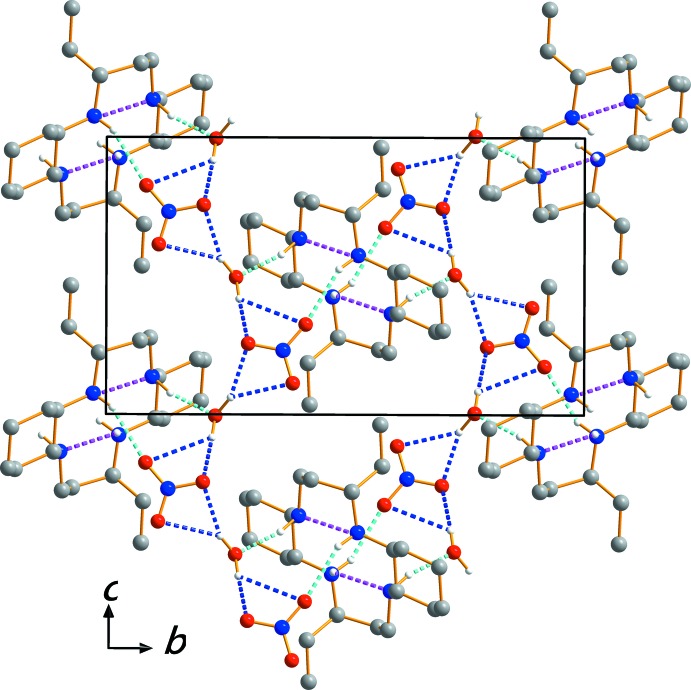
The crystal packing in (I), viewed perpendicular to the *bc* plane. Dashed lines represent N—H⋯O (cyan), N—H⋯N (pink) and O—H⋯O (blue) hydrogen bonding inter­actions, respectively. H atoms bound to C have been omitted.

Extensive N—H⋯O, O—H⋯O and N—H⋯N hydrogen-bonding inter­actions occur in the crystal structure (Table 1 ▸). The crystal packing viewed along the *a* axis is shown in Fig. 2 ▸. The O—H⋯O hydrogen bonds link the water mol­ecules to neighboring nitrate anions, while N—H⋯O hydrogen bonds inter­connect the [C_22_H_46_N_4_]^2+^ cations with both the nitrate anions and the water mol­ecules. The crystal structure is stabilized by mol­ecular hydrogen bonds involving the macrocycle N—H groups and water O—H groups as donors, and the O atoms of the water mol­ecules and nitrate anions as acceptors, giving rise to a three-dimensional framework (Figs. 1 ▸ and 2 ▸).

## Database survey   

A search of the Cambridge Structural (Version 5.40, Feb 2019 with 1 update; Groom *et al.*, 2016 ▸) gave just three hits for organic compounds containing the macrocycles [C_22_H_48_N_4_]^4+^, [C_22_H_46_N_4_]^2+^ or (C_22_H_44_N_4_). The crystal structures of [C_22_H_46_N_4_](ClO_4_)_2_ (Aree *et al.*, 2018 ▸), [C_22_H_46_N_4_]Cl_2_·4H_2_O (Moon *et al.*, 2013 ▸) and (C_22_H_44_N_4_)_2_·2NaClO_4_ (Aree *et al.*, 2018 ▸) were reported by us previously. Until now, no crystal structures of any [C_22_H_46_N_4_]^2+^ or [C_22_H_48_N_4_]^4+^ compounds with a nitrate anion have been deposited.

## Synthesis and crystallization   

Commercially available *trans*-1,2-cyclo­hexa­nedi­amine, ethyl vinyl ketone and silver nitrate (Sigma–Aldrich) were used as provided. All other chemicals were reagent grade and used without further purification. As a starting material, 3,14-diethyl-2,6,13,17-tetra­aza­tri­cyclo­(16.4.0.0^7,12^)docosane, *L* was prepared according to a published procedure (Lim *et al.*, 2006 ▸). A solution of the macrocyclic ligand, *L* (0.33 g, 1.0 mmol) in methanol 10 mL was added dropwise to a stirred solution of AgNO_3_ (0.34 g, 2.0 mmol) in water 10 mL. The solution turned an orange color and the metallic silver that formed was filtered off. The orange filtrate was kept in an open beaker, protected from light, at room temperature. Block-like colorless crystals of suitable for X-ray analysis were obtained unexpectedly from the solution over a period of a few weeks.

## Refinement   

Crystal data, data collection and structure refinement details are summarized in Table 2 ▸. All C and N-bound H atoms in the complex were placed in geometrically idealized positions and constrained to ride on their parent atoms, with C—H distances of 0.97–0.99 Å, and with an N—H distance of 0.90 Å with *U*_iso_(H) values of 1.2 and 1.5 times the *U*_eq_ of the parent atoms, respectively. The N-bound H atoms of the [C_22_H_46_N_4_]^2+^ cation and the O-bound H atoms of the water mol­ecules were located in a difference-Fourier map and refined isotropically, with the N—H distance restrained using DFIX [0.9 (2) Å] and the O—H distances and H—O—H angles restrained using DFIX and DANG constraints [0.94 (2) and 1.55 (2) Å], respectively.

## Supplementary Material

Crystal structure: contains datablock(s) I. DOI: 10.1107/S2056989019007655/sj5572sup1.cif

Structure factors: contains datablock(s) I. DOI: 10.1107/S2056989019007655/sj5572Isup2.hkl

CCDC reference: 1918729

Additional supporting information:  crystallographic information; 3D view; checkCIF report

## Figures and Tables

**Table 1:** Hydrogen-bond geometry (Å, °)

*D*—H⋯*A*	*D*—H	H⋯*A*	*D*⋯*A*	*D*—H⋯*A*
N2—H2*AN*⋯N1	0.90	2.40	2.9703 (18)	121
N2—H2*AN*⋯N1^i^	0.90	2.41	2.8141 (17)	107
N1—H1*N*⋯O4	0.94	1.84	2.7493 (19)	163
N2—H2*AN*⋯N1	0.90	2.40	2.9703 (18)	121
N2—H2*BN*⋯O1	0.90	2.27	3.031 (2)	142
O4—H1*O*⋯O1	0.94 (1)	2.57 (2)	3.169 (3)	122 (2)
O4—H1*O*⋯O2	0.94 (1)	1.84 (1)	2.768 (2)	174 (2)
O4—H2*O*⋯O2^ii^	0.94 (1)	2.04 (1)	2.914 (2)	155 (2)
O4—H2*O*⋯O3^ii^	0.94 (1)	2.31 (2)	3.120 (4)	144 (2)

Symmetry codes: (i) 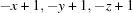; (ii) 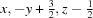.

**Table 2:** Experimental details

Crystal data	
Chemical formula	C_22_H_46_N_4_^2+^·2NO_3_^−^·2H_2_O
*M*_r_	526.68
Crystal system, space group	Monoclinic, *P*2_1_/*c*
Temperature (K)	220
*a*, *b*, *c* (Å)	8.6420 (17), 16.687 (3), 9.7340 (19)
β (°)	96.46 (3)
*V* (Å^3^)	1394.8 (5)
*Z*	2
Radiation type	Synchrotron, λ = 0.610 Å
μ (mm^−1^)	0.07
Crystal size (mm)	0.13 × 0.09 × 0.05

Data collection	
Diffractometer	Rayonix MX225HS CCD area detector
Absorption correction	Empirical (using intensity measurements) (*HKL3000sm *SCALEPACK**; Otwinowski & Minor, 1997 ▸)
*T*_min_, *T*_max_	0.919, 1.000
No. of measured, independent and observed [*I* > 2σ(*I*)] reflections	14284, 3736, 2968
*R*_int_	0.027
(sin θ/λ)_max_ (Å^−1^)	0.693

Refinement	
*R*[*F* ^2^ > 2σ(*F* ^2^)], *wR*(*F* ^2^), *S*	0.062, 0.211, 1.10
No. of reflections	3736
No. of parameters	170
No. of restraints	4
H-atom treatment	H atoms treated by a mixture of independent and constrained refinement
Δρ_max_, Δρ_min_ (e Å^−3^)	0.73, −0.56

Computer programs: *PAL BL2D-SMDC Program* (Shin *et al.*, 2016 ▸), *HKL3000sm* (Otwinowski & Minor, 1997 ▸), *SHELXT2018* (Sheldrick, 2015*a* ▸), *SHELXL2018* (Sheldrick, 2015*b* ▸), *DIAMOND 4* (Putz & Brandenburg, 2014 ▸) and *publCIF* (Westrip, 2010 ▸).

## supplementary crystallographic information

### Crystal data

**Table:**

C_22_H_46_N_4_^2+^·2NO_3_^−^·2H_2_O	*F*(000) = 576
*M**_r_* = 526.68	*D*_x_ = 1.254 Mg m^−^^3^
Monoclinic, *P*2_1_/*c*	Synchrotron radiation, λ = 0.610 Å
*a* = 8.6420 (17) Å	Cell parameters from 46866 reflections
*b* = 16.687 (3) Å	θ = 0.4–33.7°
*c* = 9.7340 (19) Å	µ = 0.07 mm^−^^1^
β = 96.46 (3)°	*T* = 220 K
*V* = 1394.8 (5) Å^3^	Block, colorless
*Z* = 2	0.13 × 0.09 × 0.05 mm

### Data collection

**Table:**

Rayonix MX225HS CCD area detector diffractometer	2968 reflections with *I* > 2σ(*I*)
Radiation source: PLSII 2D bending magnet	*R*_int_ = 0.027
ω scan	θ_max_ = 25.0°, θ_min_ = 2.0°
Absorption correction: empirical (using intensity measurements) (*HKL3000sm SCALEPACK*; Otwinowski & Minor, 1997)	*h* = −11→11
*T*_min_ = 0.919, *T*_max_ = 1.000	*k* = −21→21
14284 measured reflections	*l* = −13→13
3736 independent reflections	

### Refinement

**Table:**

Refinement on *F*^2^	4 restraints
Least-squares matrix: full	Hydrogen site location: mixed
*R*[*F*^2^ > 2σ(*F*^2^)] = 0.062	H atoms treated by a mixture of independent and constrained refinement
*wR*(*F*^2^) = 0.211	*w* = 1/[σ^2^(*F*_o_^2^) + (0.1295*P*)^2^ + 0.2454*P*] where *P* = (*F*_o_^2^ + 2*F*_c_^2^)/3
*S* = 1.10	(Δ/σ)_max_ < 0.001
3736 reflections	Δρ_max_ = 0.73 e Å^−^^3^
170 parameters	Δρ_min_ = −0.56 e Å^−^^3^

### Special details

**Table:**

Geometry. All esds (except the esd in the dihedral angle between two l.s. planes) are estimated using the full covariance matrix. The cell esds are taken into account individually in the estimation of esds in distances, angles and torsion angles; correlations between esds in cell parameters are only used when they are defined by crystal symmetry. An approximate (isotropic) treatment of cell esds is used for estimating esds involving l.s. planes.

### Fractional atomic coordinates and isotropic or equivalent isotropic displacement parameters (Å^2^)

**Table:**

	*x*	*y*	*z*	*U*_iso_*/*U*_eq_	
N1	0.51219 (14)	0.59626 (8)	0.36407 (11)	0.0308 (3)	
H1N	0.473300	0.634200	0.422800	0.037*	
N2	0.28123 (13)	0.47313 (7)	0.42499 (11)	0.0278 (3)	
H2AN	0.385711	0.476834	0.431710	0.033*	
H2BN	0.246719	0.513235	0.475356	0.033*	
C1	0.76020 (15)	0.60393 (9)	0.51374 (13)	0.0286 (3)	
H1	0.716885	0.647557	0.566781	0.034*	
C2	0.93544 (17)	0.61843 (10)	0.51640 (16)	0.0365 (3)	
H2A	0.983763	0.617356	0.612413	0.044*	
H2B	0.981148	0.575002	0.466503	0.044*	
C3	0.97155 (18)	0.69835 (10)	0.45121 (17)	0.0386 (4)	
H3A	0.936038	0.742285	0.506664	0.046*	
H3B	1.084368	0.703693	0.450342	0.046*	
C4	0.89135 (18)	0.70410 (10)	0.30389 (15)	0.0373 (3)	
H4A	0.935358	0.663904	0.245983	0.045*	
H4B	0.910323	0.757161	0.266013	0.045*	
C5	0.71640 (17)	0.69051 (9)	0.30041 (15)	0.0342 (3)	
H5A	0.667680	0.692382	0.204553	0.041*	
H5B	0.670830	0.733224	0.352056	0.041*	
C6	0.68403 (16)	0.60924 (9)	0.36398 (13)	0.0296 (3)	
H6	0.726833	0.566354	0.308993	0.035*	
C7	0.42136 (18)	0.59147 (10)	0.22379 (14)	0.0353 (3)	
H7A	0.467359	0.550519	0.168718	0.042*	
H7B	0.427202	0.643030	0.176434	0.042*	
C8	0.25211 (17)	0.57083 (9)	0.23440 (14)	0.0337 (3)	
H8A	0.192762	0.580870	0.144215	0.040*	
H8B	0.212715	0.607652	0.300777	0.040*	
C9	0.21823 (17)	0.48504 (9)	0.27811 (13)	0.0314 (3)	
H9	0.103745	0.478672	0.271539	0.038*	
C10	0.2802 (3)	0.42191 (11)	0.18588 (16)	0.0471 (4)	
H10A	0.393272	0.428242	0.189202	0.056*	
H10B	0.259978	0.368749	0.222744	0.056*	
C11	0.2090 (4)	0.42595 (16)	0.0363 (2)	0.0792 (8)	
H11A	0.096417	0.427628	0.032597	0.119*	
H11B	0.239639	0.378996	−0.012820	0.119*	
H11C	0.245453	0.473817	−0.006600	0.119*	
N3	0.2912 (2)	0.63006 (12)	0.76865 (18)	0.0581 (5)	
O1	0.3086 (2)	0.58294 (10)	0.6746 (2)	0.0812 (6)	
O2	0.2872 (2)	0.70307 (10)	0.74420 (17)	0.0696 (5)	
O3	0.2875 (5)	0.6094 (2)	0.8868 (3)	0.1636 (16)	
O4	0.42576 (16)	0.72874 (9)	0.50503 (14)	0.0501 (3)	
H1O	0.373 (3)	0.7221 (16)	0.5829 (17)	0.075*	
H2O	0.366 (3)	0.7605 (14)	0.439 (2)	0.075*	

### Atomic displacement parameters (Å^2^)

**Table:**

	*U*^11^	*U*^22^	*U*^33^	*U*^12^	*U*^13^	*U*^23^
N1	0.0327 (6)	0.0418 (7)	0.0171 (5)	−0.0017 (5)	−0.0003 (4)	0.0035 (4)
N2	0.0303 (6)	0.0363 (6)	0.0163 (5)	−0.0001 (4)	−0.0003 (4)	0.0006 (4)
C1	0.0300 (6)	0.0370 (7)	0.0185 (6)	−0.0006 (5)	0.0012 (5)	0.0007 (5)
C2	0.0303 (7)	0.0486 (9)	0.0302 (7)	−0.0013 (6)	0.0012 (5)	0.0050 (6)
C3	0.0361 (7)	0.0496 (9)	0.0305 (7)	−0.0078 (6)	0.0055 (6)	0.0016 (6)
C4	0.0380 (7)	0.0482 (9)	0.0269 (7)	−0.0029 (6)	0.0090 (6)	0.0038 (6)
C5	0.0368 (7)	0.0423 (8)	0.0240 (6)	−0.0006 (5)	0.0048 (5)	0.0065 (5)
C6	0.0317 (6)	0.0389 (7)	0.0180 (6)	0.0007 (5)	0.0024 (5)	0.0015 (5)
C7	0.0393 (8)	0.0476 (8)	0.0176 (6)	−0.0062 (6)	−0.0027 (5)	0.0057 (5)
C8	0.0344 (7)	0.0437 (8)	0.0213 (6)	0.0024 (5)	−0.0040 (5)	0.0046 (5)
C9	0.0329 (7)	0.0430 (8)	0.0172 (6)	−0.0033 (5)	−0.0013 (5)	0.0010 (5)
C10	0.0721 (12)	0.0461 (10)	0.0240 (7)	−0.0041 (8)	0.0097 (7)	−0.0044 (6)
C11	0.141 (3)	0.0743 (15)	0.0214 (8)	−0.0135 (15)	0.0046 (11)	−0.0093 (9)
N3	0.0674 (11)	0.0633 (11)	0.0418 (9)	−0.0045 (8)	−0.0025 (8)	0.0016 (7)
O1	0.1056 (14)	0.0553 (10)	0.0739 (12)	0.0074 (8)	−0.0284 (10)	−0.0228 (8)
O2	0.1060 (14)	0.0545 (9)	0.0509 (9)	0.0048 (8)	0.0204 (9)	−0.0098 (6)
O3	0.285 (5)	0.140 (3)	0.0760 (18)	0.010 (3)	0.063 (2)	0.0501 (17)
O4	0.0555 (8)	0.0574 (8)	0.0384 (7)	0.0082 (6)	0.0088 (6)	−0.0009 (5)

### Geometric parameters (Å, º)

**Table:**

N1—C7	1.4987 (17)	C5—H5B	0.9800
N1—C6	1.5009 (18)	C6—H6	0.9900
N1—H1N	0.94	C7—C8	1.517 (2)
N2—C1^i^	1.4784 (18)	C7—H7A	0.9800
N2—C9	1.4850 (16)	C7—H7B	0.9800
N2—H2AN	0.9000	C8—C9	1.531 (2)
N2—H2BN	0.9000	C8—H8A	0.9800
C1—C2	1.5309 (19)	C8—H8B	0.9800
C1—C6	1.5330 (18)	C9—C10	1.520 (2)
C1—H1	0.9900	C9—H9	0.9900
C2—C3	1.524 (2)	C10—C11	1.517 (3)
C2—H2A	0.9800	C10—H10A	0.9800
C2—H2B	0.9800	C10—H10B	0.9800
C3—C4	1.524 (2)	C11—H11A	0.9700
C3—H3A	0.9800	C11—H11B	0.9700
C3—H3B	0.9800	C11—H11C	0.9700
C4—C5	1.525 (2)	N3—O3	1.204 (3)
C4—H4A	0.9800	N3—O1	1.229 (3)
C4—H4B	0.9800	N3—O2	1.241 (2)
C5—C6	1.529 (2)	O4—H1O	0.937 (10)
C5—H5A	0.9800	O4—H2O	0.941 (9)
			
C7—N1—C6	115.09 (11)	N1—C6—C1	108.07 (11)
C7—N1—H1N	114	C5—C6—C1	110.95 (12)
C6—N1—H1N	109	N1—C6—H6	109.0
C1^i^—N2—C9	115.19 (10)	C5—C6—H6	109.0
C1^i^—N2—H2AN	108.5	C1—C6—H6	109.0
C9—N2—H2AN	108.5	N1—C7—C8	111.11 (11)
C1^i^—N2—H2BN	108.5	N1—C7—H7A	109.4
C9—N2—H2BN	108.5	C8—C7—H7A	109.4
H2AN—N2—H2BN	107.5	N1—C7—H7B	109.4
N2^i^—C1—C2	114.50 (12)	C8—C7—H7B	109.4
N2^i^—C1—C6	109.65 (11)	H7A—C7—H7B	108.0
C2—C1—C6	108.97 (11)	C7—C8—C9	116.55 (12)
N2^i^—C1—H1	107.8	C7—C8—H8A	108.2
C2—C1—H1	107.8	C9—C8—H8A	108.2
C6—C1—H1	107.8	C7—C8—H8B	108.2
C3—C2—C1	112.32 (12)	C9—C8—H8B	108.2
C3—C2—H2A	109.1	H8A—C8—H8B	107.3
C1—C2—H2A	109.1	N2—C9—C10	111.14 (12)
C3—C2—H2B	109.1	N2—C9—C8	109.38 (11)
C1—C2—H2B	109.1	C10—C9—C8	113.13 (13)
H2A—C2—H2B	107.9	N2—C9—H9	107.7
C4—C3—C2	110.74 (13)	C10—C9—H9	107.7
C4—C3—H3A	109.5	C8—C9—H9	107.7
C2—C3—H3A	109.5	C11—C10—C9	113.86 (17)
C4—C3—H3B	109.5	C11—C10—H10A	108.8
C2—C3—H3B	109.5	C9—C10—H10A	108.8
H3A—C3—H3B	108.1	C11—C10—H10B	108.8
C3—C4—C5	110.87 (12)	C9—C10—H10B	108.8
C3—C4—H4A	109.5	H10A—C10—H10B	107.7
C5—C4—H4A	109.5	C10—C11—H11A	109.5
C3—C4—H4B	109.5	C10—C11—H11B	109.5
C5—C4—H4B	109.5	H11A—C11—H11B	109.5
H4A—C4—H4B	108.1	C10—C11—H11C	109.5
C4—C5—C6	110.41 (12)	H11A—C11—H11C	109.5
C4—C5—H5A	109.6	H11B—C11—H11C	109.5
C6—C5—H5A	109.6	O3—N3—O1	123.1 (3)
C4—C5—H5B	109.6	O3—N3—O2	117.4 (2)
C6—C5—H5B	109.6	O1—N3—O2	119.23 (19)
H5A—C5—H5B	108.1	H1O—O4—H2O	109.8 (18)
N1—C6—C5	110.75 (11)		
			
N2^i^—C1—C2—C3	179.62 (12)	N2^i^—C1—C6—C5	176.50 (11)
C6—C1—C2—C3	56.43 (16)	C2—C1—C6—C5	−57.46 (15)
C1—C2—C3—C4	−55.92 (17)	C6—N1—C7—C8	174.85 (12)
C2—C3—C4—C5	55.43 (18)	N1—C7—C8—C9	−71.31 (16)
C3—C4—C5—C6	−57.05 (17)	C1^i^—N2—C9—C10	−61.66 (16)
C7—N1—C6—C5	64.03 (15)	C1^i^—N2—C9—C8	172.72 (11)
C7—N1—C6—C1	−174.25 (12)	C7—C8—C9—N2	68.87 (16)
C4—C5—C6—N1	178.64 (11)	C7—C8—C9—C10	−55.60 (17)
C4—C5—C6—C1	58.63 (16)	N2—C9—C10—C11	174.71 (16)
N2^i^—C1—C6—N1	54.91 (14)	C8—C9—C10—C11	−61.8 (2)
C2—C1—C6—N1	−179.05 (12)		

Symmetry code: (i) −*x*+1, −*y*+1, −*z*+1.

### Hydrogen-bond geometry (Å, º)

**Table:**

*D*—H···*A*	*D*—H	H···*A*	*D*···*A*	*D*—H···*A*
N2—H2*AN*···N1	0.90	2.40	2.9703 (18)	121
N2—H2*AN*···N1^i^	0.90	2.41	2.8141 (17)	107
N1—H1*N*···O4	0.94	1.84	2.7493 (19)	163
N2—H2*AN*···N1	0.90	2.40	2.9703 (18)	121
N2—H2*BN*···O1	0.90	2.27	3.031 (2)	142
O4—H1*O*···O1	0.94 (1)	2.57 (2)	3.169 (3)	122 (2)
O4—H1*O*···O2	0.94 (1)	1.84 (1)	2.768 (2)	174 (2)
O4—H2*O*···O2^ii^	0.94 (1)	2.04 (1)	2.914 (2)	155 (2)
O4—H2*O*···O3^ii^	0.94 (1)	2.31 (2)	3.120 (4)	144 (2)

Symmetry codes: (i) −*x*+1, −*y*+1, −*z*+1; (ii) *x*, −*y*+3/2, *z*−1/2.
